# Faculty’s lived experiences in recognizing nursing students’ psychological support needs in Saudi Arabia

**DOI:** 10.1186/s12909-025-07652-3

**Published:** 2025-07-15

**Authors:** Samirh Said Alqhtani, Seham Alselami, Hend Alnajjar

**Affiliations:** 1 College of Nursing, King Saud bin Abdulaziz University for Health Sciences, Riyadh, Saudi Arabia; 2 King Abdullah International Medical Research Center, Riyadh, Saudi Arabia; 3 College of Nursing, King Saud bin Abdulaziz University for Health Sciences, Jeddah, Saudi Arabia; 4 King Abdullah International Medical Research Center, Jeddah, Saudi Arabia

**Keywords:** Faculty members, Nursing students, Psychological support, Mental health, Recognition of distress, Mental health resources, Nursing education, Saudi Arabia

## Abstract

**Background/Objectives:**

Nursing students often experience high levels of stress, anxiety, and depression throughout their academic and clinical education. Academic and social pressures can intensify mental health challenges during university studies. Faculty members play a vital role in fostering a supportive and inclusive learning environment that prioritizes the mental well-being of nursing students. This study aimed to gain a deeper understanding of faculty members’ experience in recognizing nursing students’ psychological support needs.

**Methods:**

This qualitative study involved purposive sampling of faculty members in two nursing colleges in Saudi Arabia. In-depth, face-to-face, semi-structured interviews were conducted with eight faculty members using open-ended questions. The interviews were recorded, transcribed verbatim, and thematically analyzed using the NVivo program.

**Results:**

Data analysis revealed four major themes: students’ emotional support, challenges of supporting students, recognition of distress and utilizing resources, and faculty training needs.

**Conclusions:**

This study identified a multidimensional role of faculty members in supporting students’ emotional well-being and academic success. However, they faced challenges that need to be addressed through educational organizations. There is a need to examine the effectiveness of training programs in educating faculty on identifying mental health issues and providing adequate support.

**Supplementary Information:**

The online version contains supplementary material available at 10.1186/s12909-025-07652-3.

## Background

The mental well-being of nursing students is essential for their academic success, professional development, and overall quality of life. Previous research has demonstrated that nursing students frequently encounter elevated levels of stress, anxiety, and depression due to academic, clinical training, and social factors [1, 2]. In alignment with the goals of Saudi Vision 2030, the Ministry of Education has emphasized the importance of mental health awareness within academic institutions [3]. Therefore, Wellness Centers were established on most university campuses to provide psychological counseling for students. However, nursing students face academic stress due to heavy coursework, exams, and clinical training hours in Saudi Arabia [3, 4]. Mental health issues can affect students’ cognitive ability and academic success [5]. Many studies reported a high prevalence of nursing students: 65.1% had depression, and 83.19% had anxiety [6, 7].

Researchers reported the importance of assessing nursing students’ stress and early detection of depression before students experience academic failure [8, 9]. Thus, faculty members play a crucial role in creating a supportive learning environment that promotes the mental health of nursing students. They can offer emotional assistance, direction, and connections to relevant support services [10]. Positive faculty-student relationships help to reduce emotional distress and increase a sense of belonging within the academic environment [11]. However, faculty members face uncertainty and experience in providing timely support to students with mental health issues because of tensions between their academic and professional roles and the lack of explicit policies between faculty and student support services [12]. Various studies indicate a gap between faculty members’ readiness to discuss students’ mental health and the decision to refer students to mental health services [13]. Therefore, nursing faculty must be able to identify when students are stressed before it manifests in symptoms. However, research reported that faculty members had a lack of knowledge, training, or confidence to deal with a student facing mental health challenges, which can hinder their ability to recognize and support students [13].

Faculty members are on the frontline to identify at-risk students, provide information about mental health services, and refer them for help. International studies reported different strategies improved faculty’s knowledge and awareness, such as an education program [14], therapeutic communication techniques [15], and a trauma-informed training program [16] to identify and support students experiencing emotional distress. However, such initiatives are rarely implemented within the Saudi nursing education context.

Understanding the faculty members’ experiences in identifying psychological support for the students has not been explored in Saudi Arabia. Therefore, this study aimed to deeply understand the meaning of faculty members’ lived experiences in recognizing nursing students’ psychological support needs. This study is significant in identifying faculty members’ experiences in supporting nursing students, providing practical strategies, and tailoring interventions to inform faculty members and optimize students’ well-being and academic success.

## Aim of the study

This study aimed to understand the meaning of faculty members’ lived experiences in recognizing nursing students’ psychological support needs.

### Research question

What is the lived experience of faculty members in recognizing nursing students’ psychological support needs?

### Methodology

#### Design and participants

A qualitative phenomenological approach was used to explore the meaning of, gain a deeper understanding of human experiences, and influence all research processes [17]. The COREQ checklist was followed to report this study’s findings [18]. A purposive sampling strategy was used to recruit faculty members from two nursing colleges located in the Central and Western regions of Saudi Arabia. This sampling method is useful for recruiting participants who have an interest and providing rich data about the phenomenon [19]. Researchers recruited faculty members who work at the College of Nursing and are enrolled in advising roles. The exclusion criteria are staff who do not have a teaching or advising role. The number of participants recruited based on data saturation was eight. The sample size was determined when there were no new themes emerged and thematic repetition was observed [17, 20].

#### Rigor

Lincoln & Guba’s criteria were employed to ensure this study’s trustworthiness [20]. Credibility of the findings was established through prolonged engagement with the data and by providing rich, detailed descriptions of participants’ experiences. Additionally, peer debriefing allowed researchers to review and offer constructive feedback, enhancing the validity of the findings. Collaborative analyses involving multiple perspectives enriched the depth and accuracy of the results. Dependability was addressed by creating a clear audit trail. Confirmability was achieved using a reflexive journal, where the researchers consistently recorded their thoughts, reactions, and decisions throughout the study process. Transferability was enhanced by including direct quotes from participants to provide authentic and detailed insights into their experiences. The accuracy of the data was further supported by transcribing audio recordings immediately after interviews and cross-checking these transcripts against the original recordings.

### Data collection

Data were collected through face-to-face interviews. After participants signed the written consent form, the interviews were conducted. The researchers collected demographic data via questionnaire in the first 5 min of the interview. Interviews were semi-structured and used open-ended questions, which lasted 45 to 60 min. All interviews were conducted in English and tape recorded.

### Data analysis

The transcripts of interviews were imported into the NVivo version 15 software for data management and organizing the codes and themes development. A thematic analysis was used following the six-step outline by Braun & Clarke [21]. The inductive approach was used to allow themes to emerge from data without any pre-existing frameworks.

1- Familiarizing with data: the researchers immersed themselves in the data by reading and re-reading the data (interview transcripts and observational notes) to gain a deep understanding of its content.

2- Generating initial codes: Two researchers (SAA and SA) systematically generated the initial codes and organized them using NVivo. Both researchers coded the data by identifying and labeling interesting features or patterns independently. NVivo facilitated coding and organizing data segments.

3- Searching for themes: The researchers organized codes into meaningful groups to identify patterns and relationships by using coding strips in NVivo to facilitate visualization of patterns.

4-Reviewing themes: the researchers generated a thematic map by checking the themes in relation to the coded extracts and the entire data set.

5- Defining and naming themes: The researchers refined the specifics of each theme and generated clear definitions and names for each theme.

6- Producing the report: The researchers then finalized and reflected on the identified themes and related the analysis to the research question and literature.

Coding was performed independently by two researchers to ensure the reliability of the coding process. Discrepancies were resolved through regular discussion until consensus was achieved. The NVivo’s query functions calculate code frequency and percentages that enhance the transparency of the coding process Table 1.

**Table 1:** Themes coding overview

Theme	No. of Codes	No. of Faculty Mentioning Theme (out of 8)	% of Faculty
Students’ Emotional Support	28	7	87.5%
Challenges of Supporting Students	15	6	75.0%
Recognition of Distress & Resources	20	8	100%
Faculty Training Needs	28	8	100%

## Results

### Demographic data

Eight faculty members participated in this study. The demographic characteristics are presented in Table 2.

**Table 2:** Demographic characteristics of faculty members (*N* = 8)

Participants	Academic service	Number of Supervising students	Academic rank	Specialty in Nursing
P1	> 5 years	20	Lecturer	Critical Care
P2	> 5 years	10	Lecturer	Maternity
P3	> 5 years	10	Lecturer	Medical and Surgical
P4	> 5 years	10	Associate professor	Maternity
P5	< 5 years	20	Assistant professor	Community
P6	> 5 years	15	Assistant professor	Psychiatrics
P7	> 5 years	10	Assistant professor	Community
P8	< 5 years	10	Assistant professor	Management and leadership

### Themes

The data analysis revealed four major themes: “students’ emotional support,” “challenges of supporting students,” “recognition of distress and utilizing resources,” and “faculty training needs.”

### Theme 1: student emotional support

Faculty members created an empathetic, non-judgmental environment where students feel comfortable sharing their emotional struggles. Most of the participants shared a strong commitment to listening and emotional presence with different levels of confidence. Active listening was central to the faculty’s experiences in supporting students. Most faculty expressed that all students need to be heard and be present with them. This theme also reflects that faculty members provide academic advice and provide space to express their emotions, which supports students’ emotional well-being. A participant illustrated, “Sometimes, my way of communication is treating advisees differently than the students I lecture. I do not lecture them. I let them vent… they need emotional support, someone who listens to them.” (Participant 6).

Further, the participants were mindful that maintaining confidentiality is crucial to fostering trust when students are vulnerable. Most participants emphasized the importance of creating a safe environment of acceptance and trust, as illustrated, “… I closed the door and made sure that we had a private environment. I told her that she could speak and I would listen. I tried not to interrupt her” (Participant 4). However, confidence levels varied in providing emotional support, while others were uncertain, as explained in the next theme. This positions faculty members as compassionate guidance rather than fixing the students’ problems.

This theme also reflects a proactive approach. Several faculty members discussed reaching out to students showing signs of distress through direct interactions, using both in-person and online sessions. A participant said, “I approach the students during their class, which is the only way I can contact them. Sometimes, I use the Team’s program because the students like to write emails and text messages and feel more comfortable than face-to-face conversations.” (Participant 1).

In contrast, several faculty members expressed the importance of face-to-face communication for building trust and being flexible in using online communication channels to support students who might feel hesitant about face-to-face conversations. A participant stated, “… certain students say, I want to talk to you privately. Then, in those circumstances, I give them an appointment through WhatsApp or a call. They can call me, and then they come and meet me personally” (Participant 7). This flexibility in faculty helps students feel empowered and fosters an environment where they can contact faculty to seek help.

Faculty members also extended their availability time outside regular working hours to support students. This informal availability indicates recognizing students’ emotional distress, commitment, and having a deep sense of empathy. Further, easy accessibility goes beyond academic advising, showing a genuine commitment to students’ mental health and stability. A participant reported, “I follow up with students if it is a serious situation because I want to see how the student gets better. I will contact the student,… I will call them…” (Participant 2).

Faculty members expressed their role as academic guidance, supported students’ personal development, and assisted them with work-life balance. A participant explained, “I get some of the students who have low GPAs or low achievements or no grades in the course, and I will contact them to come to my office and discuss what I can do for them.” (Participant 4).

Many participants shared strategies, such as academic advice, and others used personal experiences to help students with academic struggles, such as group study sessions, personalized follow-ups, and time management. Other participants shared their own academic experiences that helped alleviate helplessness and isolation. As one stated, “. I shared my experiences with students, like my experience with public speaking,… I believe faculty members have a role in supporting students as they have been through all these things.” (Participant 1).

### Theme 2: recognition of distress and utilizing resources

This theme focuses on how faculty members identified the early signs of emotional and social distress and their collaborative role in leveraging institutional resources to address students’ needs.

Faculty members identified various indicators and used different strategies to respond to the students’ needs. Faculty members emphasized their ability to recognize visible signs of students with emotional or psychological issues through observable behavior or academic performance. Some faculty noted that academic performance is an indicator that directly impacts students’ mental and emotional well-being, such as low grades or exam difficulty. A participant illustrated, “…early detection is a huge responsibility for the faculty… if the student has an academic issue like absenteeism or something that is purely academic, not with underlying psychological issues, I handle it online. However, if I suspect, I dive deeper.” (Participant 6).

This contrasts with what other participants focused on, which was academic grades as an indicator of emotional cues. A participant stated, “Some students will not show any signs or warning signs of psychological issues, but it reflects on their grades, and we can see some issues because their assignment marks are good, but they are unable to do well in the exams.” (Participant 3).

Social withdrawal was another indicator, particularly among participants who observe students in class. Faculty members reported that isolation or withdrawal from group activities is the first sign of students’ distress. A participant stated, “I have noticed that when lecturing, it’s often apparent who is struggling, as some students may seem disengaged or isolated. These students tend to sit at the back of the class, away from others.” (Participant 4). Another said, “Sometimes, students display antisocial behaviors, such as isolating themselves. You will often see them sitting alone, not interacting with the group, or avoiding social engagement.” (Participant 2).

Faculty members also worked with course coordinators and academic affairs to coordinate efforts to address the student’s situation. A participant said, “I would create a session under the academic advisor unit. I will sit with the student, talk about her issues, and address her concerns. If it is psychological, we will do multiple sessions to address it. If unresolved, I will ask the student’s permission to communicate with the social worker.” (Participant 6).

The faculty members highlight the use of a structured approach within the college system for students in need of support and close monitoring, including advising and counseling systems and referrals to social workers or psychologists. A participant reported, “We have something called… Shawnee System or Wellness Center. Those who are specifically approaching the social workers will be maintained as separate records and monitored frequently.” (Participant 8).

Access to the available resources, such as the Wellness Center, was recognized; however, it was used based on students’ willingness. Faculty members reported that they connect students to various network resources through the advising and counseling system, such as referring to social workers or psychologists. A participant stated, “Our college offers various resources, including a wellness center with medical professionals and psychologists.” (Participant 4).

### Theme 3: challenges of supporting students

Faculty members encountered challenges in addressing students’ mental health concerns and providing adequate support. Faculty members expressed uncertainty and discomfort about using proper communication strategies to handle students’ difficulties. Although some faculty had an active and empathic role, many members reported feeling unprepared to address or assess mental health concerns and uncomfortable asking students direct questions about their mental health. A participant said, “I am not comfortable at all… if the student agrees she has psychological issues, I am kind of getting stressed because I do not know how to deal with it.” (Participant 8). A common strategy mentioned is referring students to Wellness Centers or professionals for its safest and most effective approach. A participant explained, “I prefer just to refer the student to the Wellness Center and to make sure that she is visiting the Wellness Center; this is all I can do in the situation, but it makes me feel really bad also because I could not communicate with her because I believe every word would affect her in a way or the other. So, I am very cautious about that.” (Participant 1).

This discomfort was pronounced when students did not openly disclose their struggles. Several faculty members also faced challenges with students reluctant to seek help, making it difficult for faculty to identify those needing support. These communication barriers were either due to stigma or unawareness. A participant reported, “I know about a student from her friends. They come to me and say, she is going through something. I know that she has been dealing with psychosis, but she does not like to talk about it.” These experiences reflect the tension between faculty concern and their lack of readiness in handling psychological issues.

### Theme 4: faculty training needs

This theme emphasizes the need for increased awareness of mental health resources and training for faculty members and staff on dealing with students with emotional distress. The training needs were varied among participants. Most of the faculty members discussed the need for training on handling students with psychological issues. A participant stated, “We need more workshops or education on how to detect students with mental distress and suicidal ideation.” (Participant 5).

Other faculty highlight the need for clear guidelines for mental health crises. A participant said, “We need a clear guideline or policy that I can read or know about a high case that should be reported and interact with, such as suicidal ideation.” (Participant 2). Several faculty members requested workshops and educational sessions focusing on practical skills such as communication techniques and strategies for supporting distressed students. A participant illustrated, “We need workshops about communication techniques and support techniques. I attended a workshop about giving feedback. I learned much from this session. We should have all the skills to talk with the students and give them feedback.” (Participant 3).

Some raised a concern related to students’ awareness of available resources, suggesting a clear orientation for students and staff about the availability of mental health resources, particularly the wellness center and advising units. Several participants noted that students receive guidance during orientation, but there is a lack of detailed and easily accessible information on mental health resources to seek help.

A participant explained, “…I think it should start with the orientation part where they oriented us about the university; they should give students clear pictures about the resources that we have in universities.” (Participant 4).

## Discussion

The findings of this study underscore the critical role that faculty members play in supporting both the emotional well-being and academic success of students. Faculty are in a unique position to offer both direct and indirect support to students experiencing emotional distress due to their frequent interactions, which enables them to identify and address early signs of distress. The study’s results align with previous research, particularly a study that found that faculty who actively promote mental health resources and work to reduce stigma significantly contribute to students’ well-being [22]. However, while these studies underline the positive impact of faculty engagement, there are differences in the perceived and implementation of effectiveness of these practices across different cultural and institutional contexts. In rural areas and low-resource settings, many institutions lack the infrastructure to address mental health needs and lack faculty who are trained to identify and support the students [23].

The study also emphasizes how faculty actively engage with students who show signs of distress. Their willingness to extend their availability outside of working hours and adapt various communication methods (such as online platforms) reflects a deep sense of responsibility and empathy. Such proactive engagement is essential in creating an environment where students feel accessible to faculty and can trust them enough to seek help. This dynamic contributes to a sense of belonging and community, crucial factors for fostering student motivation and engagement, particularly in high-stress academic environments [24]. However, the cultural norms and values significantly shape how support is perceived and received by students, affecting their engagement and motivation. A previous study by [25] compared the differences in educational systems between Asian and Western countries, found that in the Chinese educational system, there is greater emphasis on hierarchy and formality in the student-faculty relationship, which can hinder open communication. This hierarchical structure limits students’ autonomy and creativity. Compared to Western education, which emphasizes collaboration and independent learning, fostering a more interactive and empathetic communication style.

In contrast to these proactive strategies, it’s also worth noting that a study found that many students prefer the online personal counseling model due to its anonymity and reduced stigma [26]. Thus, integrating online counseling into existing support structures can offer comprehensive support to students in distress. Although [27] found that digital counseling platforms improved access and helped manage academic stress, the interpersonal relationship between counselors and students was weaker compared to face-to-face sessions, emphasizing the need to maintain emotional connection in digital counseling services. Thus, Further research is needed to explore the students’ perspective on the effectiveness and preferences of online counseling versus face-to-face interaction in diverse educational and cultural contexts.

Despite these positive findings, the study also revealed significant challenges that faculty face in supporting students, particularly in identifying and addressing mental health concerns. Faculty members often expressed discomfort and uncertainty about how to approach psychological issues, citing a lack of training and clear guidelines. This gap in faculty preparedness is reflected in existing research. For example, a previous study reported that faculty members lack sufficient knowledge of mental health [28] and have not received comprehensive training in crisis intervention and mental health first aid [29]. These challenges emphasize the need for institutional support to equip faculty with the necessary skills and knowledge to effectively handle such situations. Further supporting this point, research by [12] suggests that the lack of institutional support systems and clear guidelines can contribute to faculty stress and burnout. The study underscores the necessity for clear institutional policies that not only encourage open discussions of mental health with students but also help faculty develop the skills to manage cases involving severe distress or suicidal ideation. This is especially critical in addressing the broader mental health crisis in educational settings.

Additionally, the study found that faculty members were often unaware of available institutional resources, which hindered their ability to respond to student needs effectively. This finding aligns with previous studies that highlight the importance of training faculty to better understand their roles in supporting students and reducing the stigma surrounding mental health issues [12, 30, 31].

In conclusion, this study reinforces the need for comprehensive institutional support, training, and policies to empower faculty in addressing mental health challenges effectively. By fostering an informed and empathetic faculty, institutions can better support students in their academic and emotional journeys.

### Implications

The study findings highlight the need for nursing curricula to incorporate mental health awareness and emotional resilience as essential components. Nursing faculty should have clear guidelines for available institutional resources, such as wellness centers, counseling services, and social workers. Strong collaboration between nursing faculty and these resources can ensure that students receive comprehensive support. In addition to increasing awareness about mental health, it is important to ensure that students understand that seeking help is a sign of strength, not weakness, by normalizing discussions of emotional well-being. The findings of this research have several implications for nursing research.

Nursing research could focus on developing, implementing, and evaluating training programs for nursing faculty that address mental health first aid, crisis intervention, and communication strategies for engaging students in distress. This research could help assess their impact on faculty confidence and student outcomes. Furthermore, a qualitative study on understanding how faculty communication styles influence students’ emotional health could lead to the development of best practices for supporting nursing students in distress.

### Limitations

This study provides a deep understanding of the experience of faculty members recognizing nursing students’ psychological support needs. The findings of this study are limited in generalizability due to the small sample size and demographic data (gender and specialties), which are not representative of all educational contexts. Although an effort was made to include a diverse group of faculty members, based on demographic characteristics, the sample includes only female faculty members due to the limited male nursing faculty members. This study does not capture the experiences of students and their preferences for counseling methods. Thus, more research is needed on how students perceive and utilize the counseling services. A potential bias of this study is the presence of social bias that may arise due to personal beliefs to provide socially accepted responses. Additionally, a potential limitation could be related to the researcher’s bias related to the nature of a qualitative study. However, reflexivity and methodological rigor were employed in all data collection and analysis to minimize this risk.

## Conclusion

This study provides insight into faculty members’ lived experiences in recognizing nursing students’ psychological support needs. The findings emphasize the multidimensional role of faculty members in providing students with emotional and academic support. Faculty consistently demonstrate empathetic engagement by fostering a non-judgmental and confidential environment for students to share their struggles. Their efforts in connecting students to institutional resources, like wellness centers and social workers, reveal a collaborative approach to addressing mental health concerns. However, faculty encounter significant challenges, including discomfort in addressing mental health issues, communication barriers with students, and limited training in handling psychological concerns. Thus, institutional support through training, resources, and clear protocols is essential for equipping them to navigate the complexities of mental health concerns effectively.

## Electronic supplementary material

Below is the link to the electronic supplementary material.

Supplementary Material 1

## Data Availability

The full transcripts of the interviews are not available to the public due to privacy concerns. However, data from this study can be obtained from the corresponding author upon reasonable request.

## References

[1] Phu TG, Thao NH, Long PT. Stress Levels among undergraduate nursing students in relation to demographic factors at Tra Vinh university, Vietnam, 2023. *EAS J Psychol Behav Sci* 2024, *6*; 10.36349/easjpbs.2024.v06i03.001

[2] Choudhury K, Depression. Anxiety and stress among nursing students in selected colleges of Eastern india: A descriptive study. IJONE. 2024;16(1):22–6. 10.37506/4dhcwe12.

[3] Mohamed NA, Ali SO, Ehrahim EEE, Ahmed AL, Wahba AM. Predictors of academic and clinical stress among nursing students. *SAGE open nursing*. 2024 Jan *1*;10. 10.1177/23779608241290392 10.1177/23779608241290392 PMC11514110 39469726

[4] Aljohani KA. Nursing education in Saudi arabia: history and development. Cureus. 2020;12(4):e7874. 10.7759/cureus.7874. 32489728 10.7759/cureus.7874 PMC7255546

[5] Malik S. Examining cognitive functioning and academic stress in high school students: implications for education and well-being. IJSTE. 2024;12(3):2747–57. 10.22214/ijraset.2024.59339.

[6] Alreshidi SM, Rayani A, Aboshaiqah AE, Aljaloud A, Ghulman S, Alotibi A. Prevalence and associations of depression among Saudi college nursing students: A cross-sectional study. Healthcare. 2024;12(13):1316. 10.3390/healthcare12131316. 38998851 10.3390/healthcare12131316 PMC11240896

[7] Alghamdi AR. Prevalence of test-anxiety and its relationship with academic achievement: findings from nursing, physical therapy, laboratory, and radiology students in Taif city, Saudi Arabia. IJAR. 2022;10(01):150–7. 10.21474/ijar01/14024.

[8] Choudhury K. Depression, anxiety and stress among nursing students in selected colleges of Eastern india: a descriptive study. IJONE. 2024;16(1). 10.37506/4dhcwe12.

[9] Altaweel F, Kamel N, Alqahtani F. Self-esteem and determinants of depression among undergraduate nursing students in dammam, Saudi Arabia. Med Archives. 2023;77(1):44. 10.5455/medarh.2023.77.44-48. 10.5455/medarh.2023.77.44-48 PMC10008262 36919131

[10] Porter DM, Nadelson LS, Oyineyi O. Faculty members’ perspectives of supporting college students’ psychological well-being. High Educ Stud. 2025;15(1):261. 10.5539/hes.v15n1p261.

[11] Xu Y, Zhu Q, Chen Y. Teacher-student relationships and college students’ psychological well-being: the mediating role of supportive school climate. Soc Behav Pers. 2024;52(2):1–8. 10.2224/sbp.1298.

[12] Ramluggun P, Kozłowska O, Mansbridge S, Rioga M, Anjoyeb M. Mental health in higher education: faculty staff survey on supporting students with mental health needs. Health Educ. 2022;122(6):601–16. 10.1108/he-02-2022-0011.

[13] Putri AK, Saputra AR, Yahya A. Building a multi-layered support system for students in psychological distress: insights from Indonesian faculty members. Jurnal Psikologi. 2023;50. 10.22146/jpsi.80921.

[14] Lakes CT. Responding to students experiencing emotional distress: an action research study of professional learning experiences for faculty and staff. Doctoral dissertation. University of Kentucky. 2020; 10.13023/ETD.2020.131

[15] Tyson G, Beck MS. Supporting struggling students through therapeutic communication. J Christ Nurs 2021 Apr-Jun 01;*38*(2):92–7. 10.1097/CNJ.0000000000000813 10.1097/CNJ.0000000000000813 33660644

[16] Ray EC, Perko A, Oehme K, Bradley L, Arpan LM. Facilitating well-being: an examination of an online trauma-informed faculty/staff training designed to support college student resilience. Discover Educ. 2024;3(1). 10.1007/s44217-024-00199-3.

[17] Dibley L, Dickerson S, Duffy M, Vandermause R. Doing hermeneutic phenomenological research: a practical guide. London: SAGE Publications Ltd; 2020. ISBN: 978-1526485724.

[18] Tong A, Sainsbury P, Craig J. Consolidated Criteria for Reporting Qualitative Research (COREQ): A 32-item checklist for interviews and Focus Groups. *Int. J. Qual. Health Care*. 2007 Sept 16;*19*(6):349–57. 10.1093/intqhc/mzm042 10.1093/intqhc/mzm042 17872937

[19] Tappen RM. Advanced nursing research: from theory to practice. 3rd ed. Burlington (MA): Jones & Bartlett Learning; 2023.

[20] Lincoln YS, Guba EG. Naturalistic inquiry. Thousand Oaks (CA). SAGE; 1985. pp. 289–331. 10.1016/0147-1767(85)90062-8.

[21] Braun V, Clarke V. Using thematic analysis in psychology. Qual Res Psychol. 2006;3(2):77–101. 10.1191/1478088706qp063oa.

[22] Boman J, Lindsay B, Bernier E, Boyce MA. Fostering student well-being in the postsecondary teaching and learning environment. Journal Furth High Education. 2025;49(2):230–42. 10.1080/0309877x.2024.2447852.

[23] Xu DR, Samu GC, Chen J. Advancing mental health service delivery in low-resource settings. Lancet Glob Health. 2024;12(4):e543–5. 10.1016/S2214-109X(24)00031-7. 38408460 10.1016/S2214-109X(24)00031-7

[24] Luque Vilca OM, Bolivar Espinoza N, Achahui Ugarte VE, Gallegos Ramos JR. Estrés académico En estudiantes universitarios Frente a La educación virtual asociada al Covid-19. Puriq. 2022;4. 10.37073/puriq.4.1.200.

[25] Cai X. The differences and social influencing factors between contemporary Chinese and Western education systems: discussion based on teacher-student interaction. Adv Educ Humanit Soc Sci Res. 2023;5(1):281. 10.56028/aehssr.5.1.281.2023.

[26] Amri K, Karneli M, Busryo Y,. M,. S, Alam Z, Rangkuti S. R. WEB-Based [internet]-Personal counseling (e-PC) model [internet]educes anxiety in [internet]he face of National [internet]xamination. KSS [Internet]. 2023Mar.3;*8*(4):244–53. https://knepublishing.com/index.php/KnE-Social/article/view/12905

[27] Hidayat AN, Usanto U. Investigating the impact of digital counseling platforms on high school students’ well-being for enhancing emotional resilience and academic performance. Edelweiss Appl Sci Technol. 2024;8(6):720–7. 10.55214/25768484.v8i6.2143.

[28] Satparam J. Examining the mental health literacy and challenges to supporting students among regional Philippine teacher education faculty. *JEMDS.* 2023 Sept 30;*3*(3):41–53. 10.52631/jemds.v3i3.169

[29] Gunawardena H, Leontini R, Nair S, et al. Teachers as first responders: classroom experiences and mental health training needs of Australian schoolteachers. BMC Public Health. 2024;24:268. 10.1186/s12889-023-17599-z. 38263048 10.1186/s12889-023-17599-z PMC10804620

[30] 1, Sabrifha E, Darmawati D. The importance of teacher interpersonal communication as an effort to maintain students’ mental health: A study of lerature review. Jurnal Educatio: Jurnal Pendidikan Indonesia. 2022;8(2):236. 10.29210/1202222931.

[31] Zheng F. Fostering students’ Well-Being: the mediating role of teacher interpersonal behavior and student-teacher relationships. Front Psychol. 2022;12:796728. 10.3389/fpsyg.2021.796728. 35058857 10.3389/fpsyg.2021.796728 PMC8763970

