# Correction: Faculty’s lived experiences in recognizing nursing students’ psychological support needs in Saudi Arabia

**DOI:** 10.1186/s12909-025-07777-5

**Published:** 2025-08-06

**Authors:** Samirh Said Alqhtani, Seham Alselami, Hend Alnajjar

**Affiliations:** 1 College of Nursing, King Saud bin Abdulaziz University for Health Sciences, Riyadh, Saudi Arabia; 2 King Abdullah International Medical Research Center, Riyadh, Saudi Arabia; 3 College of Nursing, King Saud bin Abdulaziz University for Health Sciences, Jeddah, Saudi Arabia; 4 King Abdullah International Medical Research Center, Jeddah, Saudi Arabia

**Correction: BMC Med Educ 25, 1055 (2025)** 

**https://doi.org/10.1186/s12909-025-07652-3**

Following publication of the original article [1], the authors would like to correct the IRB number listed under the Ethics approval and consent to participate.

The sentence currently reads:

This study was conducted in accordance with the Declaration of Helsinki and approved by the Ethical Committee of King Abdullah International Medical Research Center (KAIMRC) (IRB/NRJ24/018/5).

The sentence should read:

This study was conducted in accordance with the Declaration of Helsinki and approved by the Ethical Committee of King Abdullah International Medical Research Center (KAIMRC) (IRB/NRR24/070/5).

The original article [1] has been corrected.
