# Supplementary material for: Faculty’s lived experiences in recognizing nursing students’ psychological support needs in Saudi Arabia
Source: BMC Med Educ. 2025 Jul 15;25:1055. doi: 10.1186/s12909-025-07652-3 (PMC12261848; doi:10.1186/s12909-025-07652-3)
Supplement: Supplementary file 1 — Supplementary Material 1 [file 12909_2025_7652_MOESM1_ESM.docx]

**Supplementary Information**

**Interview Questions**

1. Tell me about your experiences and how you support students who need psychological support.
2. Please describe the nature of the interaction you had with a student/s whom you believed to be affected by a mental health problem.
3. Please describe any challenges you encountered when you supported students who needed psychological support.
